# A long-term survival case of histiocytic sarcoma by surgery alone in a Japanese elderly breast tumor patient

**DOI:** 10.1186/s40792-023-01609-8

**Published:** 2023-02-28

**Authors:** Toru Higuchi, Chih-Ping Li, Yuko Hirota, Yuji Hayashi, Fumio Arisawa, Ikuko Manabe, Takashi Sakurai, Akiko Adachi, Tsuyoshi Saito

**Affiliations:** 1 Breast Unit, Japanese Red Cross Saitama Hospital, 1-5 Shintoshin, Chuoh-Ku, Saitama, 330-8553 Japan; 2 Department of Pathology, Japanese Red Cross Saitama Hospital, 1-5 Shintoshin, Chuoh-Ku, Saitama, 330-8553 Japan; 3 Department of Nursing, Japanese Red Cross Saitama Hospital, 1-5 Shintoshin, Chuoh-Ku, Saitama, 330-8553 Japan

**Keywords:** Histiocytic sarcoma, Breast tumor, Geriatric oncology, Case report, Mammography, Mastectomy, Prognosis

## Abstract

**Introduction:**

Histiocytic sarcoma (HS) is a rare hematologic malignancy. HS of the breast is extremely rare, and we present a case of an elderly patient with breast HS.

**Case presentation:**

An 81-year-old woman with unremarkable past medical and family histories presented to our hospital with a palpable mass in her right upper breast. She had noticed a mass in her right breast 3 months before her first visit. Physical examination revealed a mass measuring approximately 30 mm in the right upper quadrant of the breast; there were no cervical or axillary lymphadenopathies. Mammography revealed a high-concentration mass with unclear margins in the upper and outer breast. Ultrasound and magnetic resonance imaging (MRI) revealed a 31 × 23-mm nodule with a relatively clear margin and necrotic sign on the T2-intensified image. A mastectomy was performed upon the patient’s request, and the surgical specimen revealed a 35-mm hemorrhagic mass. The lesion was estrogen receptor-, progesterone receptor-, and HER2/neu-negative. The Ki-67 labeling index was approximately 30%. The immunohistochemical panel showed immune reactivity for the histiocytic markers CD68, CD163, and CD206 and was immune-negative for B lineage, T lineage, Langerhans cells, and keratins. The diagnosis of HS was based on the morphological and immunophenotypic characteristics of the mass. The patient received no systemic therapy and survived for 50 months without recurrence.

**Conclusions:**

Here, we report the case of an elderly patient with rare breast HS. Although the prognosis of HS seems poor, the breast HS was not as poor as expected, since it might have been discovered in the local region before it metastasized.

## Background

In Japan, the incidence of breast cancer has been increasing and is the highest among all malignant diseases [1]. Most malignant breast neoplasms are derived from epithelial cells. However, a small proportion of malignant breast tumors, such as malignant breast lymphoma, are derived from mesenchymal tissues or hematologic malignancies [2].

Malignant neoplasms derived from hematologic tissues are diverse, and histiocytic sarcoma (HS) is very rare among these entities [3]. HS originates from histiocytes and non-Langerhans cells [4]. It is diagnosed using both morphological and immunohistochemical methods. Marte et al. reported that HS constitutes less than one percent of all hematologic malignancies [4]. Patients with HS generally present with several symptoms, including fever, fatigue, weight loss, and swollen lymph nodes [2]. However, the present case only had a breast mass with none of these symptoms. We present a rare case in which breast cancer was first suspected but was eventually diagnosed as HS of the breast after a detailed examination.

## Case presentation

An 81-year-old woman with a palpable mass in the right upper breast presented to our hospital 3 months after noticing her lump for the first time. There were no swollen lymph nodes in the axilla or neck, and she had no past medical or family history of the disease. Blood counts and laboratory tests, including tumor markers, were within normal limits.

**Fig. 1:**
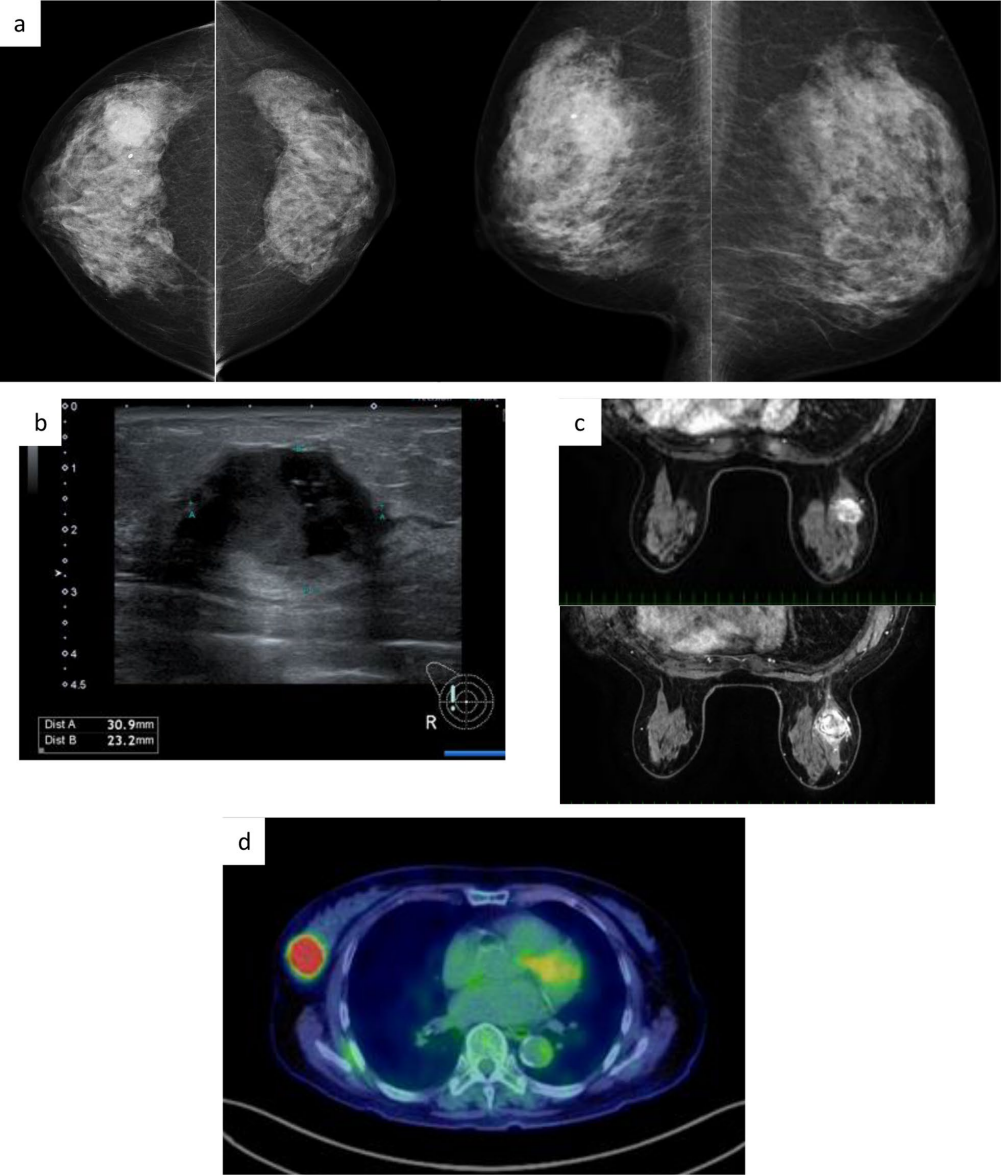
Images of the patient at the first visit. **a** Mammography: high-density mass shadow with obscure margin in the outside area of the craniocaudal outside and middle area of the mediolateral oblique. **b** Images of ultrasonography. Irregular nodule 31 × 23 mm in size. **c** Magnetic resonance imaging. Upper: early phase; Lower: late phase. Round mass with high intensity in T2 mode with rapid wash-out sign. **d** Fluorodeoxyglucose (FDG)–positron emission tomography. High concentration of FDG in the upper-outer region of the breast

Mammography showed an irregularly shaped high-density mass shadow with micro-lobulated right upper margin (Fig. 1a) and outer areas. Ultrasonography showed a well-circumscribed irregularly shaped hypoechoic lesion (Fig. 1b). The mass was heterogeneously enhanced on magnetic resonance imaging (MRI). It had intense fluorodeoxyglucose uptake on positron emission tomography–computed tomography (Fig. 1c, d), which suggested that the mass was malignant.

**Fig. 2:**
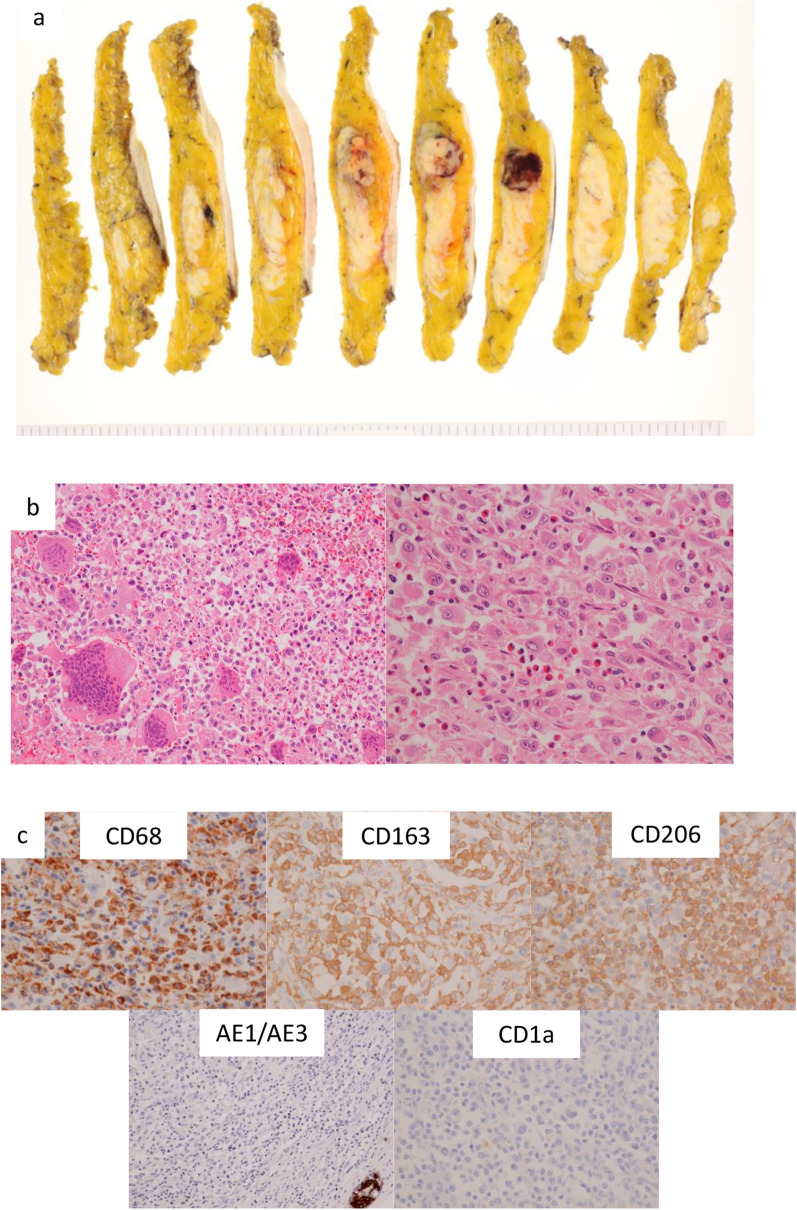
Characterization of the breast lesion. **a** Macroscopic finding. Round lesion showing well-defined margin, focal necrotic tissue, and bleeding. **b** Hematoxylin and eosin stain (HE), × 40. Proliferation of large, epithelioid cells with bizarre pleomorphic nuclei and abundant cytoplasm. HE, × 10. Prominent multinucleated giant cells showed scattered lymphocytes and eosinophils in the background. **c** Immunohistochemical panel. CD68, positive; CD163, positive; CD206, positive; AE1/AE3, negative; CD1a, negative

The histology of specimens obtained via vacuum-assisted biopsy was almost indicative of a non-carcinomatous malignant tumor but could not completely be diagnosed. Considering the possibility of the lesion being an invasive carcinoma and the patient’s eager request, we performed a mastectomy and sampling of axillary lymph nodes. Macroscopically, the surgical specimen showed a 35-mm grayish-white lesion, intermingled with bleeding, located on the right upper quadrant of the breast (Fig. 2a). Microscopically, the neoplastic cells showed large, grooved, eccentrically placed, oval nuclei with vesicular chromatin and a prominent single, irregular nucleolus. The cytoplasm was abundant and eosinophilic in nature, with numerous mitoses identified. Bleeding and necrosis were observed, as well as scattered multinucleated giant cells, lymphocytes, and eosinophils (Fig. 2b). An extensive immunohistochemical (IHC) panel was performed. The atypical cells were negative for the estrogen receptor, progesterone receptor, and HER2 receptor, and the Ki-67 labeling index was approximately 30%. Other IHC findings are summarized in Table 1. The histiocytic markers CD68, CD163, and CD206 were positive.

**Table 1:** Summary of immunohistochemistry results

**CD68**	+	aSMA	−
**CD163**	+	desmin	−
**CD206**	+	AE1/AE3	−
**S-100 protein**	+	EMA	−
**CD31**	+	HMB45	−
**CD4**	+	CD45	−
**vimentin**	+	CD3	−
CD1a	−	CD20	−
langerin	−	BRAF	−
CD30	−	**Ki67**	** + (30%)**

Bold means that the protein was diagnosed as ‘positive’ by immunohistochemical test

S-100 cells showed focal positivity. The tumor cells were negative for the epithelial markers AE1/AE3, dendritic cell markers CD1a and Langerin, and mesenchymal markers desmin and SMA. Based on the morphology and immunohistochemistry results, the tumor was diagnosed as HS (Fig. 2c). No lymph node metastasis was detected.

The postoperative course was uneventful, and the patient was discharged on the 5th postoperative day. Considering the patient’s wishes and lack of validated evidence, we followed the patient up without systemic treatment. The patient survived for 50 months after the surgery without recurrence.

## Discussion

HS is a rare hematolymphoid neoplasm that very rarely occurs in the breast. Although one case was reported in South Korea [5], this is probably the first report of breast HS in a Japanese patient [2]. To our knowledge, this is the first report of primary HS of the breast in a patient in their 80 s.

HS is extremely rare among neoplasms of hematolymphoid tissue, with a frequency of less than 1% [2, 3, 6]. The age range of onset is wide, and HS is slightly more common in men. Most patients develop B-cell or T-cell lymphoblastic lymphoma or leukemia. HS most commonly originates from the lymph nodes; the other onset sites are extranodal, including the gastrointestinal tract, spleen, soft tissues, and skin [6]. In this case, no significant changes were observed in the blood test, and a breast mass was the only finding, which is very uncommon.

The systemic symptoms of HS include fever, tiredness, night sweats, and weight loss. None of these symptoms occurred in the present case. In a previous report, there was no axillary lymphadenopathy in the primary breast and no systemic symptoms [5]; they seem to be detected in the early phase of HS before distant metastasis occurs. Although the prognosis of HS is generally poor [2, 7], breast HS seems to have a better prognosis, as elderly patients survive for many years without systemic treatment [5]. However, since this suggestion is based only on the reports of two cases, it will be necessary to accumulate and verify the cases.

In the present case, an excisional biopsy was necessary for the final diagnosis of HS. According to previous reports, cases with prominent symptoms, including fever or tiredness at the first visit, had onset from hematopoietic tissue [2]. However, cases in which HS had developed from non-hematopoietic tissue had few symptoms. The tumor could not be diagnosed during the first biopsy. Non-epithelial neoplasms, such as diffuse large B-cell lymphoma, are rarely reported as primary hematologic neoplasms of the breast [2]. HS should also be considered a differential diagnosis when non-epithelial tissue is sampled from a breast mass.

IHC staining is helpful in HS diagnosis [2]. Regarding the validation of HS diagnosis, diagnostic criteria include positivity for even one histiocytic marker and negativity for dendritic cell lineage markers, bone marrow cell/T cell/B cell lineage markers, and mesenchymal markers [6]. In detail, the diagnosis of HS requires positivity for CD163, CD68 (KP1 and PGM1), and lysozyme; and negativity for B cell- and T cell-lineage markers, typical Langerhans/Langerhans cell markers (CD1a, Langerin), follicular dendritic cells (CD21, CD23, CD35), epithelial markers (pan-cytokeratin), melanocytes (HMB-45, Melan A), and myeloid cells (CD13, CD33, myeloperoxidase). Histiocytic markers expressed in HS include CD4, CD11c, CD14, CD15, CD43, CD45, CD45RO, MAC387, and HLA-DR [6]. There is no clear standard for the Ki-67 labeling index. CD30, a marker for Hodgkin’s lymphoma, was negative. S-100, a marker for melanocytes and Langerhans cells, may be positive; however, staining is often uneven, weak, and focal. CD31, a marker of vascular endothelial cells, was locally positive. Table 1 shows the results of the IHC analysis, which were compatible with the diagnostic criteria for HS.

Anti-cancer drug treatment could be omitted because of performance status or the individual’s wishes. Although thalidomide and chemotherapy followed by autologous bone marrow stem cell transplantation have been reported to be highly effective [8], a standard treatment has not yet been established for HS [6]. In this case, adjuvant systemic therapy was not administered, because there was no distant metastasis, and the patient was old. In addition, in a report from South Korea [5], the breast HS that developed in the elderly might not need adjuvant chemotherapy. It will be difficult to establish strong evidence because of the rarity of this condition and because elderly patients are mostly excluded from clinical trials [9, 10]. Therefore, these cases are considered extremely important.

A sentinel lymph node biopsy was not performed. We wondered whether this tumor was primary breast cancer, as there were no distant metastases, including to the axilla. In addition, omitting the node examination did not seem to impair the patient’s outcome because of the patient’s natural prognosis. Mastectomy was selected considering patients’ choice, the risk of pneumonia induced by radiation after breast conservation surgery, and little contribution of radiation to improving prognosis in elderly patients [11].

## Conclusions

Here, we report a rare case of HS presenting as a breast mass. This patient survived for 50 months with only excisional treatment, suggesting that a breast-primary HS tumor without distant metastasis could be cured by only surgery. More evidence is necessary to establish the treatment for this elderly patient with a breast mass.

## Data Availability

All data generated during this study are included in the published article and its Additional files.

## Abbreviations

FDG: Fluorodeoxyglucose
HE: Hematoxylin and eosin
HS: Histiocytic sarcoma
IHC: Immunohistochemical
MRI: Magnetic resonance imaging
